# Right hemicolectomy with D3 lymph node dissection for right-sided transverse colon cancer using the Senhance robotic system: a case report

**DOI:** 10.1186/s40792-020-01037-y

**Published:** 2020-10-07

**Authors:** Atsuko Kataoka, Yasumitsu Hirano, Hiroka Kondo, Satoshi Shimamura, Masahiro Kataoka, Masahiro Asari, Takatsugu Fujii, Shintaro Ishikawa, Toshimasa Ishii, Shigeki Yamaguchi

**Affiliations:** Department of Gastroenterological Surgery, Saitama Medical University International Medical Center, 1397-1 Yamane, Hidaka, Saitama 350-1298 Japan

**Keywords:** Complete mesocolic excision, Laparoscopic surgery, Oncology, Robotic surgery

## Abstract

**Background:**

The evolution of remote systems and artificial intelligence technology has led to increase in robotic surgeries. One system used in this case report is the Senhance robotic system. The most important premise for using robotic surgery in cancer therapeutics is to ensure oncological safety. Similar to conventional laparoscopic surgery, robotic surgery needs to be a reliable and secure surgical procedure, such as complete mesocolic excisions with central vascular ligations in Western countries or D3 lymph node dissections (dissection of the lymph nodes that locates from the origin to the terminal branch of the main feeding artery of cancer) in Japan.

**Case presentation:**

A 76-year-old man underwent clinical examination for severe anemia. He was diagnosed with transverse colon cancer of tumor (T)3, node (N)1a, metastasis (M)0 cancer stage IIIA. A right hemicolectomy with D3 lymph node dissection using the Senhance surgical system was performed. The operative time was 313 min and the estimated blood loss was 5 ml. He was discharged from our hospital 12 days after the surgery without any complications. What is the remarkable of this report, not only mobilization of right colon but also D3 lymph node dissection and vascular ligation were performed intraperitoneally by using Senhance robotic system as conventional laparoscopic surgery. We tried using fourth robotic arm to accomplish lymphadenectomies and middle colic artery dissection. A right hemicolectomy with D3 dissection using the Da Vinci surgical system was reported. Another report of a right hemicolectomy performed with the Senhance robotic system was identified; however, in that study, lymph node dissections were not performed intraperitoneally.

**Conclusions:**

Therefore, to our knowledge, this is the first report using the Senhance robotic system for right hemicolectomy with D3 dissection. We hope that our case report will assist in the establishment of this robotic procedure in surgical practice.

## Background

The Senhance System (TransEnterix Surgical Inc., Morrisville, NC, USA) is a robotic surgical system that was originally developed by an Italian company, SOFAR S.P.A., and marketed as “TELELAP Alf-x”. This system is composed of three robotic arms and a cockpit. The surgeon sits in the cockpit and operates robotic arms while watching the surgical field on a three dimensional (3D) display. Since the surgeon looks at an unfixed display, stress on the back is relieved. This robotic system has other novel characteristics, including haptic feedback control and infrared-ray eye-tracking, to easily change operating fields and surgical controls. This system enables the surgeon to perform physical and functional stress-free surgeries. Although this robotic surgery is not used worldwide, the technology behind remote systems and artificial intelligence (AI) technology have made rapid advances in recent years.

D3 dissection surgical procedures (dissection of the lymph nodes that locates from the origin to the terminal branch of the main feeding artery of cancer) are standard for patients with clinical stage II and III colon cancer in Japan [1], whereas complete mesocolic excisions (CMEs) with central vascular ligations (CVLs) are widely used to treat colon cancer in Western countries; two procedures based on sound oncologic principles. Expert surgeons using both techniques have reported impressive outcomes compared with standard surgeries [2]. Colectomies using this system were first reported by Spinelli [3] in 2017, and 83 cases have been reported so far [3–14]. Although some reports using CME with CVLs or D3 dissections to treat right-sided colon cancer with the Da Vinci Surgical System (Intuitive Surgical Sunnyvale, CA, USA) were found, no reports that performed D3 lymph node dissection and vascular ligation intraperitoneally by using Senhance robotic systems. In this report, we discuss a patient that underwent a right hemicolectomy with D3 dissection for right-sided transverse colon cancer using the Senhance System.

## Case report

A 76-year-old man presented for severe anemia (hemoglobin, 4.6 g/dL) on clinical examination. On a previous examination, a cause for the anemia was assessed, a blood transfusion was performed. The colonoscopy revealed a tumor at the hepatic flexure. Well-intermediate differentiated tubular adenocarcinomas (tub1–tub2) were detected with biopsies. The patient was referred to our hospital for surgery, was 160 cm, 47.3 kg, with a body mass index (BMI) of 18.5 kg/m^2^. The past medical history was unremarkable. On presentation, the electrocardiogram and respiratory function were normal. The Eastern Cooperative Oncology Group performance status (PS) was 0, and the American Society of Anesthesiologists performance status (ASA-PS) was 1. Biochemical data obtained at the time of the patient’s first visit were as follows: white blood cell, 4.2 × 10^3^/μL (normal, 3.3–8.6 × 10^3^/μL); hemoglobin, 12.9 g/dL (14–17 g/dL); albumin, 4.0 g/dL (4.1–5.1 g/dL); C-reactive protein, 0.262 mg/dL (0.00–0.14 mg/dL); carcinoembryonic antigen (CEA), 4.6 ng/mL (0.0—5.0 ng/mL); carbohydrate antigen 19–9 (CA19-9), 11.6 U/mL (0–37 U/mL). Another colonoscopy was performed that revealed that a 3.5 cm type 2 tumor at the hepatic flexure. Tumor infiltration to the subserosal layer was suspected. A computerized tomography (CT) scan revealed a tumor at the hepatic flexure with metastasis to lymph nodes along the right branch of the middle colic artery (MCA). Distant metastases were not detected. The patient was diagnosed with transverse colon cancer of tumor (T)3, node (N)1a, metastasis (M)0 cancer stage IIIA by The Union for International Cancer Control (UICC) TNM Classification of Malignant Tumors, 8th edition.

**Fig. 1:**
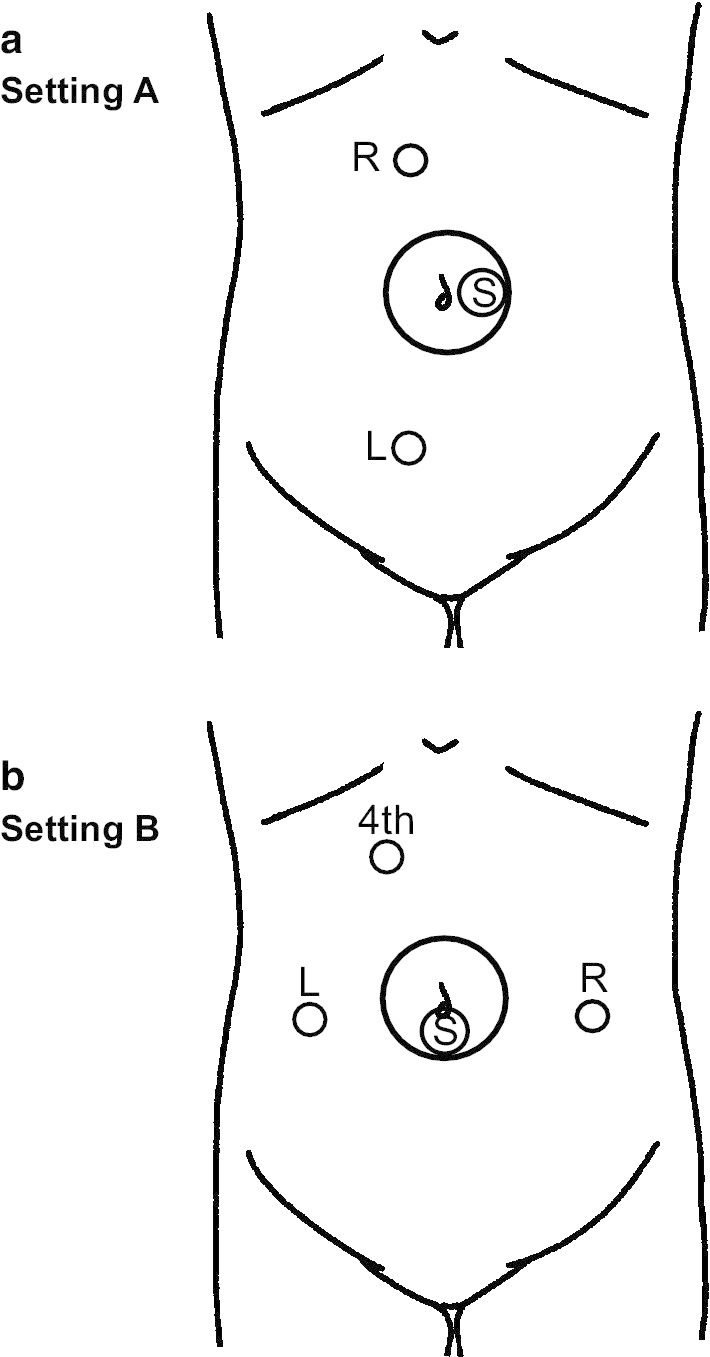
Port placement of a right hemicolectomy using the Senhance robotic system. **a** Setting A. Lymphadenectomy and dissection of the ileocolic artery (ICA) and vein (ICV) with mobilization of the right colon and separation of the outer side of the ascending colon from the retroperitoneum was performed using this port placement. **b** Setting B. Lymphadenectomy and dissection of the middle colic artery (MCA) was performed using this formation. The port for the fourth arm was inserted to expand the transverse mesocolon. Changing the port placement made it easy for the surgeon to identify the MCA by viewing the transverse mesocolon from the front

**Fig. 2:**
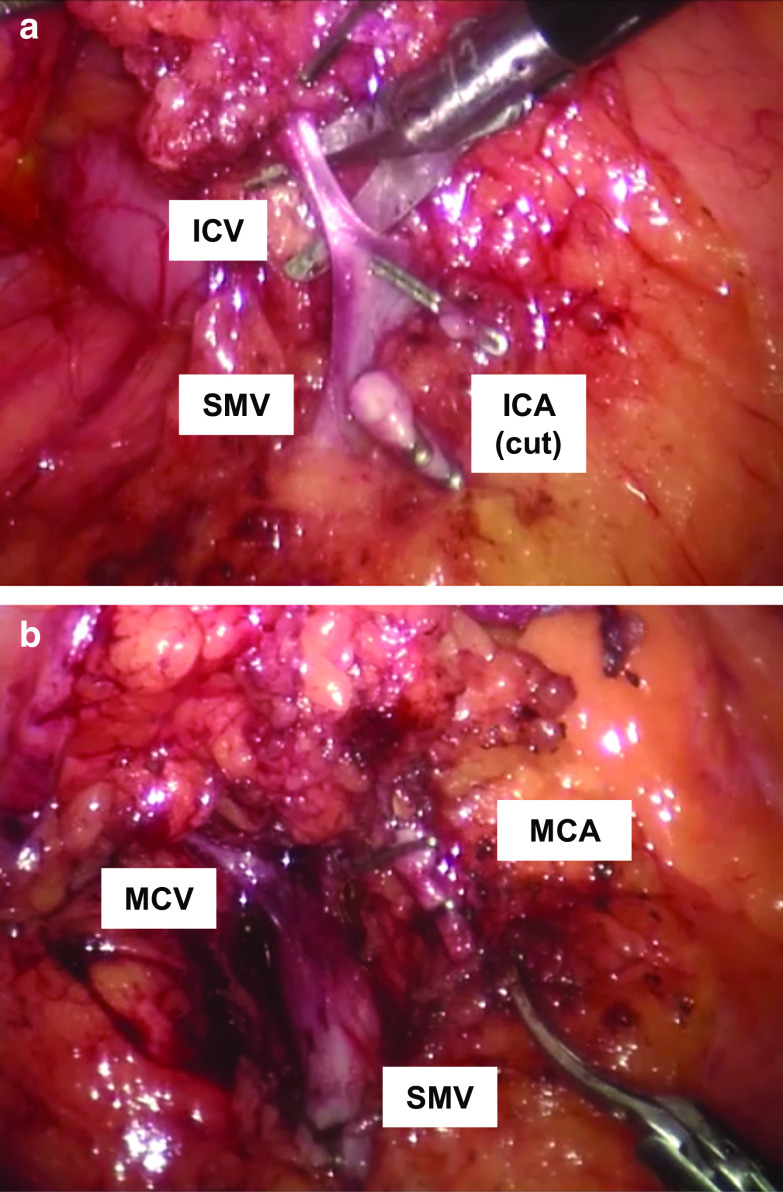
Macroscopic findings of the lymphadenectomy and vessels. **a** A photograph shows the root of the ileocolic vein (ICV) that joins the superior mesenteric vein (SMV) after lymphadenectomy. **b** A photograph shows the origin and root of the middle colic artery (MCA) after lymphadenectomy

A right hemicolectomy with D3 lymph node dissection using the Senhance surgical system was adopted. The patient’s body was placed in a lithotomy position and fixed with headgear, and both lateral side support apparatuses. First, an Alexis wound protector XS (Alexis®, Applied Medical, Rancho Santa Margarita, CA, USA) was inserted through a 2.5 cm trans-umbilical incision protecting the wound. Next, a Free Access® (TOP Corporation, Tokyo, Japan) was mounted onto the wound protector with one camera port. A 5 mm port was also available to help the surgeons during difficult situations or in an emergency. Two 5 mm ports were inserted a little to the right of center in the upper and lower abdomen (Setting A, Fig. 1). A pneumoperitoneum (pneumoperitoneum pressure: 10 cmH_2_O) was induced, and the body position was changed with a head-down tilt, and the left side was placed in a lower position. The transverse colon was flipped cranially, and the jejunum and ileum were shifted left and lateral to the abdomen and pelvis. The pedicle of the ileocolic artery (ICA) and vein (ICV) was held and pulled ventrally, and the mesentery was cut below the pedicle to mobilize the right colon. The origin of ICA was exposed using the robotic instrument. To clip the ICA, we removed the robotic instrument from the abdominal cavity and manually applied the clips with an endoscopic multiple clip applier. The ICA was clipped at the origin and divided. In this case, because the right colic artery was present, we clipped and divided this artery in the same way. Subsequently, the ICV was clipped and divided with at its root after the dissection of lymph nodes at the root of the ICV. Next, the right colon was mobilized cranially, and the outer side of the ascending colon and the root of mesoileum were separated from the retroperitoneum. After the transverse colon was pulled caudally, the hepatocolic ligament was cut and removed. Then, the mobilization of the right colon was finished. At this point, 5 mm ports were inserted on both sides of the umbilicus (Setting B, Fig. 2) to dissect the lymph nodes along the MCA. The fourth arm of the Senhance system was inserted into the upper abdominal port to spread the transverse mesocolon. The mesentery of the right colon was dissected along the anterior wall of the superior mesenteric vein (SMV). The MCA was identified and clipped at its root after the dissection of lymph nodes, performed with the two ports inserted into each lateral side of the umbilicus. We subsequently divided the MCA. The middle colic vein was also clipped and divided at its root. The gastrocolic trunk was exposed completely; however, the patient lacked an accessory right colic vein. After separating the transverse mesocolon from the omentum, the right colon was pulled outside of the abdomen. The tumor was detected on the right side of the transverse colon and the surgical specimen was removed. A functional end-to-end anastomosis was performed extracorporeally using staplers. The operative time was 313 min, the docking time was 18 min, and the console time was 196 min. The estimated blood loss was 5 mL. The patient was allowed food starting on the third postoperative day and was discharged from our hospital 12 days after the surgery without any complications. The pathological diagnosis was pT3 N0 (0/33) M0, pStage IIA. The proximal and distal margins of the lesion were free of tumor cells.

## Discussion

Hohenberger et al. [15] introduced CMEs with CVLs as standardized surgical techniques for colon cancer. CMEs with CVLs for colon cancer removes significantly more tissues around the tumor including maximal lymph node clearance that could provide superior oncologic outcomes and survival advantages compared with standard lymphadenectomies [16, 17]. Japanese surgeons have performed similar procedures known as D3 lymph node dissections which are equivalent to CMEs in terms of the radical nature of the surgical procedures. In recent years, several minimally invasive surgeries, including robotic surgeries, have been performed. When using these new surgical methods in colon cancer patients, it is important to secure oncologic safety by performing complete CMEs with CVLs or D3 dissections.

Because the Senhance robotic system has not yet been discovered worldwide, the surgical procedures have not been established. However, remote systems and AI technology have made great strides in medicine. It will not be long before surgeons can perform laparoscopic surgeries far from the operating room or that surgical robots can operate autonomously. The Senhance robotic system is one of a pioneer in the evolution of remote systems and AI technology and has the potential to become more widely used.

Spinelli et al. [3] and Samalavicius et al. [7, 9] reported on right hemicolectomies to treat colon cancer using the Senhance robotic system. In the first study, 14 colon cancer cases (including two neuroendocrine tumor cases) underwent right hemicolectomies, and although all patients had R0 resections with 100% disease-free margins, and the lymphadenectomies were adequate, no mention of CMEs or D3 dissections were given [3]. Another study showed a video vignette of one patient who underwent a right hemicolectomy for colon cancer [7]. Although CMEs were performed, the Senhance robotic system was only used to mobilize right colon via a lateral to medial approach. A lymphadenectomy, vascular ligation, intestinal resection, and anastomosis were performed using the extracorporeal method. Accordingly, in this study, we reported the novel case of a patient who underwent a right hemicolectomy with D3 dissection for transverse colon cancer using the Senhance robotic system.

Regarding postoperative complications, Spinelli et al.[3] reported on the results of the Clavien–Dindo classification. Of 23 patients that had received right hemicolectomies for colon cancer and other diseases, eight patients (34.8%) had Grade I and II complications, and two patients (8.7%) had Grade III complications. Grade III complications included one patient with was mesocolic bleeding, and therefore, the robotic surgery was converted to conventional laparoscopic surgery. In another patient, anastomotic leakage was found. Samalavincius et al.[9] reported on seven patients that had received right hemicolectomies to treat colon cancer and other diseases. However, in that study, no postoperative complications occurred. The incidence of conventional laparoscopic right hemicolectomy postoperative complications has been reported to be from 5.0% to 16.7% [18, 19]. Since the definition of postoperative complications was different in each article, we were unable to compare the incidence of postoperative complications between surgeries using the Senhance robotic system and those using conventional laparoscopy.

The most challenging elements of laparoscopic right hemicolectomies are lymphadenectomies and dissection of MCA. Transverse mesocolon expansions and MCA identifications are needed to dissect lymph nodes and the MCA. In conventional laparoscopic surgery, an assistant expands the transverse mesocolon. In Senhance robotic surgery, the surgeon operates three robotic arms including a scope. In this study, we used a novel, fourth arm from the upper abdominal port to expand the transverse mesocolon. Moreover, port arrangements changed when performing the lymphadenectomy and the dissection of MCA. The surgeon used the upper abdominal port for the right hand and lower abdominal port for the left hand, and a medial approach was made to mobilize the right colon. For the lymphadenectomies and dissection of MCA, ports were inserted into both sides lateral to the umbilicus so that the surgeon could view the transverse mesocolon from the front.

## Conclusions

In this study, we described the novel use of the Senhance robotic system to perform a right hemicolectomy and D3 dissection in a patient with colon cancer.　Additional investigations and the accumulation of cases are needed to prove safety and oncologic certainty when using the Senhance robotic system to treat colon cancer. We hope that this case report will lead to the establishment of robotic surgeries using the Senhance system for right hemicolectomies and D3 dissections.

## Data Availability

Data sharing is not applicable to this article because datasets were not analyzed for this case report.

## Abbreviations

AI: Artificial intelligence
ASA-PS: American Society of Anesthesiologists performance status
BMI: Body mass index
CA19-9: Carbohydrate antigen 19-9
CEA: Carcinoembryonic antigen
CME: Complete mesocolic excision
CT: Computerized tomography
CVL: Central vascular ligation
ICA: Ileocolic artery
ICV: Ileocolic vein
MCA: Middle colic artery
PS: The Eastern Cooperative Oncology Group performance status
SMV: Superior mesenteric vein
UICC: The Union for International Cancer Control
3D: Three dimensional
